# Biomedical PEVA Nanocomposite with Dual Clay Nanofiller: Cytotoxicity, Mechanical Properties, and Biostability

**DOI:** 10.3390/polym13244345

**Published:** 2021-12-12

**Authors:** Tuty Fareyhynn Mohammed Fitri, Azlin Fazlina Osman, Eid M. Alosime, Rahimah Othman, Fatimah Hashim, Mohd Aidil Adhha Abdullah

**Affiliations:** 1 Faculty of Chemical Engineering Technology, Universiti Malaysia Perlis (UniMAP), Arau 02600, Malaysia; tutyfareyhynn@gmail.com (T.F.M.F.); rahimah@unimap.edu.my (R.O.); 2 Biomedical and Nanotechnology Research Group, Center of Excellence Geopolymer and Green Technology (CEGeoGtech), Universiti Malaysia Perlis (UniMAP), Arau 02600, Malaysia; 3 King Abdulaziz City for Science and Technology (KACST), P.O. Box 6086, Riyadh 11442, Saudi Arabia; alosimi@kacst.edu.sa; 4 BioSES Research Interest Group, Faculty of Science and Marine Environment, Universiti Malaysia Terengganu, Kuala Nerus 21030, Malaysia; fatimah.h@umt.edu.my; 5 School of Fundamental Science, Universiti Malaysia Terengganu, Kuala Terengganu 21030, Malaysia; aidil@umt.edu.my

**Keywords:** poly(ethylene-vinyl acetate) (PEVA), nanocomposite, dual nanofiller, nanoclays, cytotoxicity, biomedical

## Abstract

Poly(ethylene-vinyl acetate) (PEVA) nanocomposite incorporating dual clay nanofiller (DCN) of surface modified montmorillonite (S-MMT) and bentonite (Bent) was studied for biomedical applications. In order to overcome agglomeration of the DCN, the S-MMT and Bent were subjected to a physical treatment prior to being mixed with the copolymer to form nanocomposite material. The S-MMT and Bent were physically treated to become S-MMT(P) and Bent(pH-s), respectively, that could be more readily dispersed in the copolymer matrix due to increments in their basal spacing and loosening of their tactoid structure. The biocompatibility of both nanofillers was assessed through a fibroblast cell cytotoxicity assay. The mechanical properties of the neat PEVA, PEVA nanocomposites, and PEVA-DCN nanocomposites were evaluated using a tensile test for determining the best S-MMT(P):Bent(pH-s) ratio. The results were supported by morphological studies by transmission electron microscopy (TEM) and scanning electron microscopy (SEM). Biostability evaluation of the samples was conducted by comparing the ambient tensile test data with the in vitro tensile test data (after being immersed in simulated body fluid at 37 °C for 3 months). The results were supported by surface degradation analysis. Our results indicate that the cytotoxicity level of both nanofillers reduced upon the physical treatment process, making them safe to be used in low concentration as dual nanofillers in the PEVA-DCN nanocomposite. The results of tensile testing, SEM, and TEM proved that the ratio of 4:1 (S-MMT(P):Bent(pH-s)) provides a greater enhancement in the mechanical properties of the PEVA matrix. The biostability assessment indicated that the PEVA-DCN nanocomposite can achieve much better retention in tensile strength after being subjected to the simulated physiological fluid for 3 months with less surface degradation effect. These findings signify the potential of the S-MMT(P)/Bent(pH-s) as a reinforcing DCN, with simultaneous function as biostabilizing agent to the PEVA copolymer for implant application.

## 1. Introduction

Polymeric materials are increasingly used in the field of biomedicine, including in the manufacture of implants. In comparison ceramic and metal materials, polymeric materials possess better flexibility, ease of shaping, and processing. The foremost necessities of the implantable medical devices for tissue protection and comfort of the patient are biocompatible, flexible, and soft [1,2]. However, only a few polymers can fulfil this pre-requisite to be employed for biomedical applications, especially the one classified as ‘biomaterial’, where it needs to interact and have close contact with biological systems. These days, many researchers are looking for novel biomaterials, particularly elastomeric materials, that can mimic soft tissue [3,4,5]. In choosing the material to be implanted in the human body and that can last for a long period, the reliability and biostability of the polymer are very essential. 

Studies on the use of polymer/clay nanocomposites for biomedical applications are gaining more attention nowadays. Most of the polymer/clay nanocomposites have been developed for hydrogel and drug delivery applications [6]. For instance, Maeda et al. [7] studied poly(ethylene glycol)-b-poly(d,l-lactide-co-glycolide) (PEG-b-PLGA) diblock copolymers/laponite nanocomposite for thermal-responsive hydrogel application. The PEG-b-PLGA possessed high solubility in water due to the presence of laponite. By using the solution mixing process, the PEG-b-PLGA was settled on the surface of the laponite platelets, triggering the thermo-responsive connection in the copolymer nanocomposite system [7]. Rapacz-Kmita et al. [8] investigated polylactide (PLA) nanocomposite films incorporating drug-intercalated montmorillonite for drug delivery application. The antibacterial activity occurred due to drug intercalation in the MMT. Interestingly, through the interaction of the drug with the MMT, PLA did not adversely affect this anti-bactericidal activity [8]. Until now, very few studies have been done on the development of polymer/clay nanocomposite for implant applications. Andriani et al. [9] explored the potential of thermoplastic polyurethane (TPU) incorporating high aspect ratio fluoromica and low aspect ratio hectorite for implant applications. Both types of nanoclays were dual-modified with two quaternary alkyl ammonium salts with differing degrees of polarity in order to improve the biocompatibility between the nanofiller with the soft segment and hard segment of the TPU matrix. As a result, improvement in the biostability of the host TPU was realized due to reduced exposure of the susceptible-to-degradation ether groups to the oxidative agents. However, no cytotoxicity data were reported in this paper [9]. Up to the present, silicone elastomer has been used extensively in the making of implantable medical devices such as for insulation of heart pacemaker devices, and breast and cochlear implants. The most commonly used silicone elastomer is polydimethylsiloxane (PDMS) [10]. However, the production of PDMS-based implant insulation film requires curing and post-curing processes. The crosslinked PDMS elastomer is not recyclable and possesses high surface tack. The mechanical properties and biostability of the PDMS biomaterial are also of particular concern for long term implant applications. Although the nanoclay can be added to enhance the mechanical performance of the PDMS matrix, unfortunately, the crosslinking process is desirable to form chemical bonds among adjacent polymer chains in order to achieve the demanded physical–mechanical properties and also rubber elasticity. Based on previous research by Meng et al. [11], the PDMS/montmorillonite-chlorhexidine acetate (PDMS/OMMT) nanocomposite films were successfully produced by establishing intercalation of montmorillonite through the solution mixing process. Outstanding mechanical properties were verified when the nanoclay content employed was lower than 0.5 mass%. Nevertheless, during the sample preparation of the polymers, the crosslinker (tetraethyl orthosilicate) was added for the crosslinking process to be initiated [11]. However, once crosslinked, the resulting thermoset silicone cannot be remelted and redissolved again. Furthermore, this silicone elastomer nanocomposite requires curing and post curing processes that give drawbacks in terms of cost and time [12]. By considering the above-mentioned drawbacks, we are investigating poly(ethylene-vinyl acetate) (PEVA) copolymer for possible use as insulation of the implantable device as an alternative to silicone elastomeric material. PEVA is biocompatible, flexible, and soft and can operate the same as elastomeric materials. Nevertheless, its advantage over thermosetting elastomer/rubber is that it can be melt processed and recycled like a thermoplastic. PEVA copolymer comprises monomer units that are linked via free radical addition polymerization throughout the double bonds of the two monomers, which are semi-crystalline polyethylene (PE) and amorphous vinyl acetate (VA) [13,14]. In this research, the PEVA with 40 wt% of vinyl acetate (VA) content was employed due to its higher flexibility [15]. The higher the composition of the VA, the more rubbery and flexible PEVA can be achieved [13,15]. This flexibility and softness are ideal properties for the material used as insulant or encapsulant of the electrically active biomedical devices. In addition, the toughness property is also important to avoid tearing and mechanical failure of the encapsulant during the long term used in the human physiological environment [16,17,18]. 

This work is proposed to study the effect of using dual clay nanofiller (DCN) in the PEVA matrix to further enhance the mechanical and biostability performance of the biomedical copolymer in order to develop a new biomaterial for long-term implantable medical devices. Nanoclays used in this study were surface modified montmorillonite (S-MMT) and bentonite (Bent). They are classified as layered silicate material, having intergallery held together by electrostatic and Van der Waals forces [6]. This feature promotes the formation of face-to-face platelet stacking (tactoids). The nanoclay is extremely difficult to disperse and align in the host polymer due to the Van der Waals forces that lead to the agglomeration of the nanoclay [19]. In order to allow good nanoclay (S-MMT and Bent) platelet exfoliation and dispersion within the copolymer matrix during the melt compounding process, loosely packed tactoids were the aim. In this research study, the physical treatment process was introduced to gain better exfoliation and dispersion and strong interfacial interaction in order to enhance the load transfer across the S-MMT/Bent/PEVA matrix interface. Through this strategy, the mechanical properties and biostability of the PEVA nanocomposite can be improved. 

A different method of physical treatment was applied for Bent and S-MMT due to the different characteristics of both nanoclays. Bent was used in its pristine (polar) form (without surface modification). Therefore, the physical treatment was done by pH control and the salt addition method to destabilize the strong interlayer forces of the clay. Acid treatment was performed (until pH 4 was reached) in order to allow repulsion of the diffuse double layer in the Bent structure [20]. Then, when salt (NaCl) was added, an electric double layer was also formed on the surface of the Bent due to the presence of free Na^+^ ions [21]. When the thickness of the +ve double layer increases, the ions spill over the edge of the clay. Thus, a face-to-edge association occurs when the +ve edge site is attracted to the −ve surface [20,21]. Consequently, changes in the association of the silicate platelets occur (in which face-to-face stacking turned into face-to-edge stacking), leading to the weakening of the Van-der Walls and electrostatic forces [20,21]. More loosely packed tactoids were obtained. In the case of S-MMT, physical treatment was done by using another approach. The presence of the non-polar organic surface modifier on the S-MMT surface requires the swelling method through mechanical action rather than ion manipulation. Magnetic stirring agitates the water medium to obtain well-mixed S-MMT/water composition prior to a swelling process by ultrasonication. During ultrasonication, the basal spacing increases due to the strong hydrodynamic shear-force and high-speed impinging liquid jets offered by the sonicator probe. The ultrasound propagates in the form of attenuated waves and enters the highly stacked nanoclay layers (tactoids). As a result, the tactoids are peeled off, becoming loosely packed nanolayers [22].

Both physically treated nanoclays were combined and applied as DCN in the PEVA nanocomposite system. Those physical treatment methods were applied to the S-MMT and Bent, respectively, to optimize their dispersion and improve their homogeneity and distribution in the matrix of the PEVA during the production of the PEVA-DCN nanocomposite. It is fully understood that if the nanofiller is not well-dispersed in the polymer matrix, deterioration of mechanical and biostability performance of the matrix occurs [23,24]. According to the previous research by Chiu et al. [25], the water vapor penetration can be reduced by the existence of nanoparticles that result in tortuous paths for water molecule diffusion. For barrier coatings, inorganic particulate nanomaterials that are commonly applied are silicon oxide (SiO_2_), aluminum oxide (Al_2_O_3_), and titanium oxide (TiO_2_) [25].

The reason to use Bent(pH-s) as co-nanofiller with the S-MMT(P) was to improve the durability of the biomedical PEVA by reducing its degradation process through interactions between the susceptible-to-degrade polyvinyl acetate phase (PVA) with the Bent(pH-s) nanofiller. This is because previous studies proved that the degradation of the PVA chains did not successfully retard when only a single nanofiller (S-MMT) was used as a nanofiller [26]. It was hypothesized that the use of the Bent(pH-s) as a co-nanofiller with the S-MMT(P) can reduce the ‘catalytic’ effect caused by the presence of the organic surfactant in the structure of the S-MMT(P) nanofiller by reducing the amount of the S-MMT(P) used as reinforcing nanofiller in the PEVA nanocomposite system. Furthermore, there will be a greater shielding for breaking of the PVA chains through the developed interaction between the PVA and the Bent(pH-s). However, to ensure that this dual S-MMT(P)/Bent(pH-s) nanofiller is safe to be used for the production of the biomedical PEVA-DCN nanocomposite, a cytotoxicity study must be performed. 

According to the literature, there is only limited research on the development of the PEVA-nanoclay based nanocomposites for biomedical application [6,26,27]. Particularly, the effect of dual S-MMT(P)/Bent(pH-s) nanofillers on the mechanical properties of the PEVA with 40 wt% VA composition is still unknown, and the best S-MMT(P):Bent(pH-s) ratio to produce the optimized biomedical PEVA nanocomposite system needs to be determined. Moreover, high VA content (40 wt% of VA composition in PEVA matrix) would benefit in flexibility and softness, but no study has revealed how this high VA content might affect the biostability of the copolymer. 

In this study, a thorough investigation of the PEVA nanocomposite system incorporating dual S-MMT(P)/Bent(pH-s) nanofiller was performed to examine its viability for the encapsulant material of the biomedical implant. Biocompatibility of the dual nanofiller was first determined through fibroblast cell cytotoxicity assay analysis. The optimum ratio of the S-MMT(P):Bent(pH-s) was determined through mechanical and biostability tests. Morphological analysis by SEM and TEM was also performed to support the results.

## 2. Materials and Methods

### 2.1. Materials

The PEVA copolymer comprised of vinyl acetate (VA) and polyethylene (PE) monomers in the ratio of 2:3 was supplied by Euroscience Sdn. Bhd (Kuala Lumpur, Malaysia) and manufactured by Sigma-Aldrich (St. Louise, MO, USA). The surface modified montmorillonite (S-MMT), which contains long chains surfactant of dimethyl dialkyl (C14-C18) amine, was employed as a nanofiller, and it is known as Nanomer (types 1.44P) by Nanocor (Hoffman Estate, IL, USA) and supplied by Sigma-Aldrich (St. Louise, MO, USA). Natural bentonite clay (Bent) from China was supplied by Multifilla (M) Sdn. Bhd. (Selangor, Malaysia) and was used as a co-nanofiller in this study. Nitric acid (HNO_3_) 65% concentration by MERCK (Darmstadt, Germany) and sodium chloride (NaCl) by Sigma-Aldrich (St. Louise, MO, USA) were supplied by A.R. Alatan Sains (K) Sdn. Bhd. (Alor Setar, Malaysia). Both chemicals were used in the physical treatment process of the natural bentonite (by pH control and salt addition).

### 2.2. Preparation of Nanofillers

#### 2.2.1. Physical Treatment by Magnetic Stirring and Ultra-Sonication of Surface Modified Montmorillonite (S-MMT(P))

The physical treatment process of the S-MMT was prepared in a water medium (ratio of distilled water:S-MMT = 1:1). Then, a magnetic stirrer was employed to homogenize the mixture into suspension. This process was done for 2 h at room temperature. After that, the suspension was ultrasonicated for 5 min using a Branson Digital Ultrasonic Disruptor/Homogenizer (Model 450 D) supplied by ProSciTech (Queensland, Australia). Next, the resultant supernatant was placed onto the filter paper to remove the distilled water, and the filtered semi-dry powder was subsequently placed into the oven at 50 °C for 48 h for the drying process. Finally, the resultant powder, the so called physically treated S-MMT (S-MMT(P)), was ground and sieved.

#### 2.2.2. Physical Treatment by pH Control and Salt Addition of Bentonite(Bent(pH-s))

Firstly, the Bent suspension was prepared by mixing 20 g Bent with 100 mL of distilled water. Next, the suspension was tip-sonicated for 15 min by using the ultrasonic probe. By dropping HNO_3_, the pH of the suspension was adjusted until it reached pH 4. Then, the tip-sonication process was continued for another 5 min. Next, the mixture was added with NaCl (0.01 M) and stirred for 5 min. After that, the suspension was washed with hot water (80 °C) and underwent a drying process at 80 °C for 24 h. Finally, the dried sample of physically treated Bent by pH control and salt addition (Bent(pH-s)) was grounded and sieved.

### 2.3. Preparation of PEVA Nanocomposites and PEVA-DCN Nanocomposites

The neat PEVA, PEVA nanocomposite, and PEVA nanocomposite with dual clay nanofiller (PEVA-DCN nanocomposite) with a varied ratio of S-MMT(P):Bent(pH-s) (4:1, 3:2, 2:3, 1:4) were prepared by the melt compounding technique. The formulation and acronyms of the PEVA nanocomposites and PEVA-DCN nanocomposites are summarized in Table 1. The melt compounding process was done at 130 °C with a 50 rpm screw speed by using an internal mixer (Brabender plasticoder) machine manufactured by GmbH & Co. KG (Kulturstr, Duisburg, Germany). Firstly, the PEVA copolymer pellets were fed into a feeder. The copolymer was allowed to melt in the mixer. It took around 4 min for the copolymer to fully melt before the nanofillers could be added. Then, the melt compounding between the matrix and filler was performed for about 6 min. Next, the sample was compressed using a compression molding machine, model GT-7014-H30C by GOTECH Co. (Taichung City, Taiwan), to produce a nanocomposite sample in sheet form (130 °C) through 5 min of pre-heating, 3 min of pressing, and 10 min of cooling time. Lastly, the specimens in the form of sheets were cut according to their measurements for characterization and testing.

### 2.4. Fourier Transform Infrared Spectroscopy (FTIR)

The chemical functional groups of the pristine S-MMT, S-MMT (P), pristine Bent, and Bent(pH-s) were determined through FTIR analysis. The test was performed using a Paragon 1000 FTIR spectrometer (Perkin Elmer) equipped with Attenuated Total Reflectance (ATR) (Waltham, MA, USA). The spectra were collected in the wavenumber range of 4000 cm^−1^–650 cm^−1^, a scan number of 16, and resolution of 4 cm^−1^.

### 2.5. X-Ray Diffraction (XRD)

The samples of nanofillers before and after the physical treatment (S-MMT vs. S-MMT(P), Bent vs. Bent(pH-s)) were analyzed using a high resolution Rigaku Mini Flex II Diffractogram (Tokyo, Japan) X-ray diffractometer. The testing was carried out at room temperature with an angle range (2*θ*) from 2° to 10° at a scanning rate of 1° min^–1^. XRD was implemented in determining the change in the basal spacing (*d*) of the S-MMT and bentonite nanofiller before and after the physical treatment. This was to prove that the physical treatment by magnetic stirring and ultra-sonication of S-MMT and physical treatment by pH control and salt addition of Bent processes successfully increased the basal spacings of both nanoclays before being used as nanofiller. The Bragg’s Law formulation in Equation (1) was used to connect between the diffraction angle and basal spacing (*d*):

(1) nλ=2dsinθ

where

*λ* = wavelength of the rays;
*θ* = angle between the incident rays and the surface of the crystal;
*d* = spacing between the clay layers.

### 2.6. Fibroblast Cell Cytotoxicity Assay (Biocompatibility Test)

Since the biomedical PEVA nanocomposite is to be implanted in the human biological systems, assessing the biocompatibility of the DCN is essential. For this purpose, the MTT metabolic assay protocol was performed to measure the fibroblast cell cytotoxicity using -[4.5-dimethlthizol-2-yl]-,5-diphenyltetrazolium bromide. The cytotoxicity level of the sample was measured based on a fifty percent inhibition concentration (IC_50_) value [28,29]. First, the fibroblast cells were seeded in a 96-well microplate at 2.5 × 10^5^ well/cells and went through an incubation process in 5% CO_2_ at 37 °C. Then, the medium was taken out after 24 h and was substituted with the medium that comprised the samples, which were the pristine and physically treated nanofillers (S-MMT, S-MMT(P), Bent, and Bent(pH-s)) to over a range of doubling dilutions of 0–100 μg/mL. For each treatment, triplicate cultures were established. After 72 h, the 20 μL of MTT (5 mg/mL) in phosphate buffered saline (PBS) solution was added to each well. The incubation process took place for another 4 h in the CO_2_ incubators. Then, removal of the remaining supernatants was executed, continued by the addition of the 150 μL of DMSO into each well. The mixture was stirred thoroughly to dissolve the formazan crystal that formed in the well. In addition, the incubation process was done for a few minutes to ensure that all crystals were completely dissolved. Next, the cytotoxicity effect of the samples on the fibroblast cells was observed using the microplate reader (TECAN (Infinite M200) (Morrisville, NC, USA) by measuring the absorbance of each well at 570 nm. The values of IC_50_ stand for test agent concentration that decreases the mean of cell viability of the untreated wells to 50%. This is important to ensure the reproducibility of results. The significant difference between various concentrations in the treatments was compared by using GraphPad prism 7.01 statistical package at a 95% confidence interval (CI).

### 2.7. Tensile Test (Ambient)

The tensile test was performed by using an Instron machine model-5582 (Norwood, MA, USA), according to the ASTM D638 method. The samples were the neat PEVA, PEVA nanocomposites, and PEVA-DCN nanocomposites with varying S-MMT(P):Bent(pH-s) nanofiller ratios. The samples were turned into dumbbell shapes by punching them using the ASTM D-638-M-5 die. The testing was carried out by applying a crosshead speed of 50 mm/min for rigid and semi-rigid material. The mean values of tensile strength, toughness (area under the stress–strain curve), Young’s modulus, and elongation at break of each sample were recorded based on the measurement of five replicates. The in vitro tensile test was performed to measure the biostability of the selected samples, which are neat PEVA, PEVA-S, and PEVA-B (control samples) and an optimum sample of PEVA-DCN nanocomposite (PEVA-S_4_B_1_).

### 2.8. Transmission Electron Microscopy (TEM)

Dispersion of the nanofiller in the PEVA matrix was analyzed using transmission electron microscopy (TEM)(JEOL JEM2010 electron microscope (Japan)), operating at 200 kV. TEM analysis was performed on the control samples and the optimized PEVA-DCN nanocomposite only (PEVA-S_4_B_1_). The samples were cut at an approximately 300 nm thickness by using a diamond cutter on a Leica Ultracut Ultramicrotome (UCT) instrument at Tg temperature = −80 °C retained by employing liquid nitrogen. By using a drop of 2.5 M sucrose solution, the sample was picked up to the 200 mesh Cu grid. Before viewing, the samples were air dried under a covered petri dish. The images of each sample were taken at low magnification (10,000×) and high magnification (30,000×).

### 2.9. Scanning Electron Microscope (SEM)

The tensile fractured surfaces of the neat PEVA, PEVA nanocomposites and PEVA-DCN nanocomposites were imaged using SEM (model JEOL JSM-6010LV) (JEOL. Ltd., Tokyo, Japan). The image of each sample was taken at 500× magnification. For the samples that were subjected to the in vitro tensile test, the sign of degradation upon the hydration process was also analyzed through the morphology of the fractured surface. Before viewing under the SEM, all the sample specimens were coated by using a JFC-1600 Auto Fine Coater (JEOL Ltd., Tokyo, Japan) with platinum.

### 2.10. Biostability Analysis

The neat PEVA, PEVA nanocomposites (PEVA-B and PEVA-S), and PEVA-DCN nanocomposite (PEVA-S_4_B_1_) samples were subjected to in vitro conditions for 3 months before being subjected to tensile test and surface degradation analysis for the biostability evaluation. The in vitro treatment involved the immersion of samples under phosphate buffered saline (PBS) solution at 37 °C as the simulated body fluid. The in vitro tensile testing was carried out under similar experimental parameters/conditions as the ambient tensile tests. The data (before and after in vitro exposure) were compared through the results of the in vitro tensile test and surface morphology captured by SEM to detect degradation effects on the samples after long in vitro exposure. 

## 3. Results and Discussion

### 3.1. Characterization and Biocompatibility Analysis of the Physically Treated DCN (S-MMT(P)/Bent(pH-s))

The pristine S-MMT and Bent and their physically modified form (S-MMT(P) and Bent(pH-s)) were characterized using Fourier transform infrared analysis (FTIR) and X-ray diffraction analysis (XRD). Furthermore, the cytotoxicity assay test was performed to examine the biocompatibility of these nanofillers.

#### 3.1.1. Fourier Transform Infrared Analysis (FTIR)

FTIR analysis was done to differentiate the chemistry/structure of the S-MMT and Bent (before and after the physical treatment processes). This is important to ensure that the physical treatment by magnetic stirring and ultra-sonication process would not cause detachment of the organic surfactant on the surface of the MMT and there would be no leftover acid and alkali in the structure of the Bent upon physical treatment by the pH control and salt addition method.

**Figure 1:**
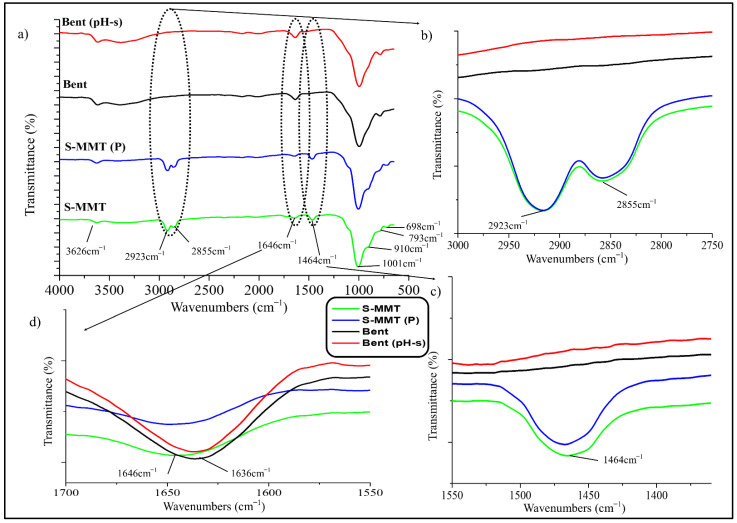
FTIR spectra of the pristine S-MMT, physically treated S-MMT (S-MMT(P)), pristine Bent and physically treated Bent (Bent(pH-s)) in a scan range (**a**) 4000 cm^−1^–500 cm^−1^; (**b**) 3000 cm^−1^–2750 cm^−1^; (**c**) 1550 cm^−1^–1360 cm^−1^; (**d**) 1700 cm^−1^–1550 cm^−1^.

Figure 1 shows the FTIR spectra of the S-MMT, S-MMT(P), Bent, and Bent(pH-s) nanofillers. Based on the data obtained, there is no major difference between the spectra of the pristine and the physically treated nanofillers. This signalized that both S-MMT and Bent did not go through massive chemistry or structure modification upon the physical treatment processes.

Furthermore, there was no significant difference between the spectra of both silicate materials (S-MMT vs. Bent). This is because both nanofillers are under the group of smectite clay, which belongs to the 2:1 phyllosilicates family in which one octahedral sheet is sandwiched between two tetrahedral sheets (T:O:T) [21,23,30,31]. For instance, both nanofillers possess a peak that emerged at ~3626 cm^−1^ due to the stretching of the cations and hydroxyls groups from the octahedral sheet [32].

The spectra peaks at ~2923 cm^−1^ and ~2855 cm^−1^, which appeared in the IR signal of the S-MMT nanofiller, indicate the existence of organic surfactant that related to intermolecular attractions between the adjacent alkyl chain (dimethyl dialkyl amine) in the S-MMT inter-galleries, while the peak at ~1464 cm^−1^ was attributed to the quaternary ammonium salt that produced the vibration through the –CH_2_ bending mode. This indicates the intercalation of the organic surfactant molecules between the silicate layers in the S-MMT nanofiller. However, these three peaks (~2923 cm^−1^, ~2855 cm^−1^, and ~1464 cm^−1^) were absent in the spectra peak of Bent (Figure 1b,c). Hence, this proved that the Bent nanofiller did not undergo surface modification with any organic compound. The bands at ~1646 cm^−1^ (S-MMT) and ~1636 cm^−1^ (Bent) were due to the bending in-plane vibration of the hydroxyl group. For Bent, a broad band also appeared at ~3389 cm^−1^ due to hydroxyl stretching vibration assigned to the adsorption of the water molecules on the clay surfaces. Furthermore, the presence of water molecules could also be confirmed by the deformation peak at ~1646 cm^−1^ to ~1636 cm^−1^ (Figure 1d) [32,33]. These peaks were more intense in the IR signal of Bent when benchmarked with those of S-MMT. This was due to the hydrophilic characteristics of the Bent nanoclay.

The most intense band, which appeared at ~1001 cm^−1^, was associated with the stretching vibrations of the Si-O group, located in tetrahedral silica sheets. The bands at ~910 cm^−1^ and ~880 cm^−1^ could be attributed to Al-Al-OH and Al-Fe-OH of bending vibration, respectively [34], while the IR bands at ~793 cm^−1^ and ~698 cm^−1^ represented the quartz admixtures present in the sample. A more intense band at ~793 cm^−1^ for Bent was attributed to a platy form of disordered tridymite, while a strong band at ~698 cm^−1^ for S-MMT was due to quartz content [32,35].

The results proved that the IR signals of both the S-MMT and S-MMT(P) are almost similar, indicating that the physical treatment by magnetic stirring and ultra-sonication processes does not cause any alteration of the chemistry aspect of the nanoclay due to the unwanted event such as leaching of the organic surfactant. In this study, it is important to ensure that the organic surfactant does not leach out from the surface of the MMT because it may bring a cytotoxicity effect to the PEVA nanocomposite. Furthermore, the Bent also was proven to retain its chemistry aspect after the pH control and salt addition processes. This was expected to happen because both physical treatments only involved physical modification of the nanoclays, not chemical modification.

#### 3.1.2. X-ray Diffraction Analysis (XRD)

XRD was implemented in determining the change in the basal spacing of the S-MMT and Bent nanofiller before and after the physical treatment processes. This was to determine whether the applied methods altered the S-MMT and Bent interlayer spacing. It is understood that if the interlayer spacing of the nanofiller increases, it will allow greater intercalation of the PEVA copolymer chains in between the nanoclay’s interlayers, thus allowing for better nanofiller dispersion in the structure of the PEVA matrix. In this study, the diffraction peaks at 2*θ* angles of 2° to 10° were recorded from the XRD signals to allow for a clear and more accurate comparison, especially at a basal spacing of *d*_001_ of all nanofillers.

**Figure 2:**
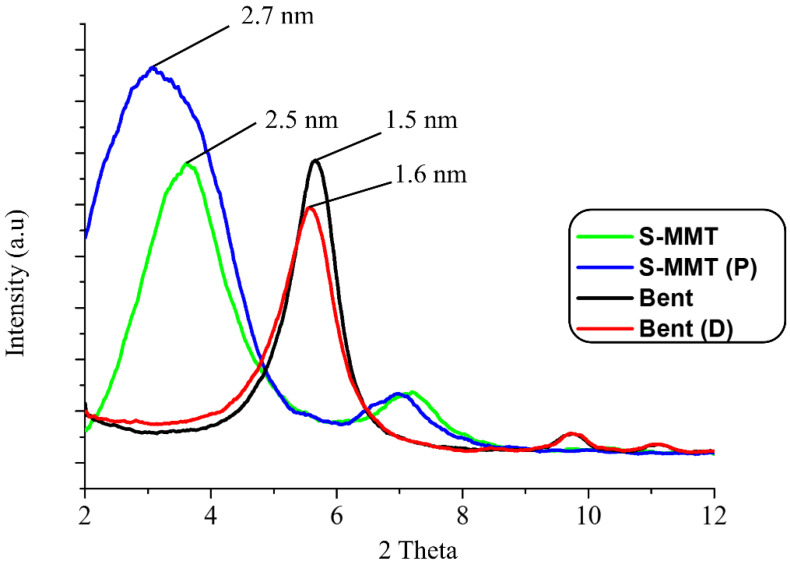
X-ray diffraction patterns of pristine S-MMT, S-MMT(P), pristine Bent, and Bent(pH-s).

The XRD patterns of the S-MMT, S-MMT(P), Bent, and Bent(pH-s) are illustrated in Figure 2. The pristine S-MMT showed *d*_001_ and *d*_002_ basal spacing of 2.5 nm and 1.2 nm, respectively, whereas after the physical treatment processes, the nanofiller (S-MMT(P)) displayed larger basal spacing for *d*_001_ and *d*_002_, which were 2.7 nm and 1.3 nm, respectively. This points out that the basal spacing of the S-MMT slightly increased after the physical treatment by magnetic stirring and ultra-sonication processes due to the swelling of the nanoclay platelet [30].

According to Hamid et al. [22], the ultrasonication process of the S-MMT in a water medium (S-MMT:H_2_O = 1:1) could cause disorientation/tilting/misalignment of the clay layered structure, resulting in the loosening of the tactoids of the MMT. This is the reason why an increment in the basal spacing is obtained. Basically, the magnetic stirring only agitates the liquid to speed up the mixing of H_2_O and S-MMT. Remarkably, when combined with the ultra-sonication process, the basal spacing increases due to the strong hydrodynamic shear-force and high-speed impinging liquid jets that are produced by the ultrasonicator. Through the growth of small vacuum bubbles which are known as ‘cavitation’ (the formation of bubbles in liquid), the ultrasound propagates in the form of attenuated waves and the entering of highly stacked nanoclay layers (tactoids). As a result, the tactoids are peeled off, becoming loosely packed nanolayers.

On the other hand, the pristine Bent showed a basal spacing (*d*_100_) value of 1.5 nm (Bent), but after undergoing the physical treatment (Bent(pH-s)), the value increased to 1.6 nm. Basically, the Bent has a pH-dependent charge on the edge of the clay and a permanent negative charge on the clay surface. The physical treatment process that involved the combination of pH control (pH 4) and salt addition (NaCl) method was an efficient technique for enhancing the basal spacing (*d*_001_) of bentonite nanoclay. This is because the exposure of Bent to the electrolytic and acidic dispersing environment led to the modification of the Bent’s silicate layer arrangement and association. This reduced the interlayer charge and surface energy of the clays to allow improvement in the degree of swelling [19]. As a result, an increment in the basal spacing could be seen.

Apparently, the basal spacing (*d*_100_) value of the S-MMT was higher compared to Bent. This is due to the presence of the bulky organic surfactant chains that intercalate the interlayers of the S-MMT. In contrast, there is no organic surfactant in the interlayers of the Bent [36].

#### 3.1.3. Fibroblast Cell Cytotoxicity Assay

**Figure 3:**
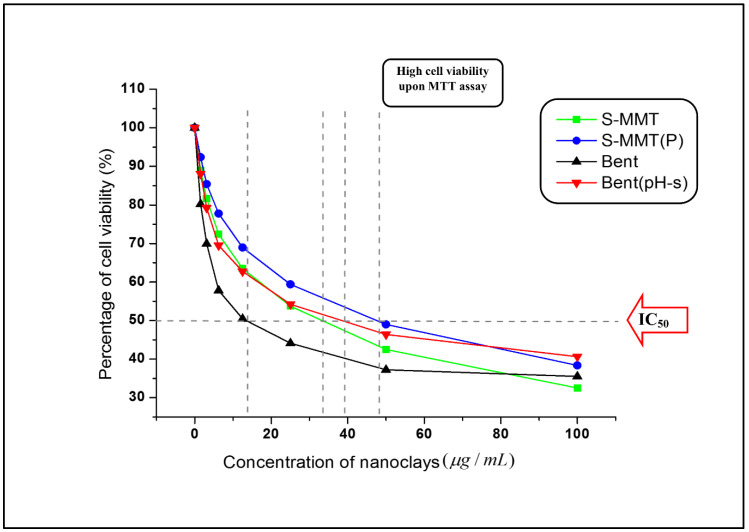
Cytotoxicity determination of S-MMT, S-MMT(P), Bent, and Bent(pH-s).

Results indicate that both nanoclays (S-MMT and Bent) cause cell death in a concentration-dependent manner [37,38]. However, types of nanoclay and physical treatment affect the inhibitory concentration (IC_50_) value, which refers to the concentration of the nanoclay suspension required to kill 50% of the living cells. The testing rules for the in vitro cytotoxicity determination suggested that the cytotoxicity potential towards the cell culture can be generated when the IC_50_ of the test agents is lower than 30 μg/mL [28,29]. Based on Figure 3, we can conclude that except for Bent, all the samples would probably lead to mild cytotoxicity against the fibroblast cell line as all the IC_50_ values obtained were above 30 μg/mL [39,40]. Interestingly, the percentage of cell viability was higher in the S-MMT(P) and Bent(pH-s) as compared to the S-MMT and Bent, respectively, meaning that the cell survival was enhanced by the physical treatment processes of the nanoclays. Particularly for Bent, the IC_50_ value increased drastically from 13.79 μg/mL (Bent) to 39.28 μg/mL (Bent(pH-s)), suggesting that the original form of Bent with a fairly high cytotoxicity level reduced to a mild cytotoxicity level only. On the other hand, the IC_50_ of the S-MMT was enhanced from 33.54 μg/mL to 48.00 μg/mL (S-MMT (P)) after the nanoclay underwent the physical treatment.

Even though such a result was obtained, the actual cytotoxicity level of both S-MMT(P) and Bent(pH-s) when being used as dual nanofiller in the PEVA nanocomposite system could be much lower because (1) the DCN is embedded in the matrix of PEVA copolymer, and thus there will be a very low possibility to induce cytotoxicity; and (2) the maximum composition of S-MMT(P)/Bent(pH-s) being used is 4 wt% only from the overall nanocomposite composition. This is a very low concentration as opposed to the IC_50_ of both nanoclays.

Whenever the cytotoxicity of the nanoparticle is mentioned, the issue of reactive oxygen species (ROS) is also always raised. ROS is a sort of unstable molecule that comprises oxygen and without difficulty reacts with other molecules in the cell. The existence of ROS in cells may affect the destruction of protein, RNA, DNA and may lead to cell death [41]. When ROS disturbs the balance between oxidative pressure and antioxidant defense, oxidative stress occurred [42]. The ability to cause oxidative stress is one of the major mechanisms that could initiate adverse biological responses that lead to toxicological effects of the nanoparticles, including the nanoclays. According to the previous research studies, nanoclay with agglomerated and larger tactoids tend to damage the cell membrane by forming intracellular ROS. Consequently, cell damage occurs through the formation of localized oxidative stress. Lordan et al. [42] and Jeevanandam et al. [43] stated that the aggregation, size, shape, and composition of the nanofiller in the cell culture medium affect their interactions with the biological systems and hence the cytotoxicity mechanisms. Basically, the agglomeration and reactivity of nanoparticles are dependent on their particle size. When the particle size is smaller, the agglomeration will occur at slower rates, and hence the cell membrane damage will be reduced. This is the reason why the nanoclay with a delaminated/exfoliated structure can cause lower cell death in the biological system. In other words, tactoid size reduction and delamination reduce the cytotoxicity level of the nanoclay. This is in line with our results that show that by using the nanoclay with more loosely packed structure/broken tactoids (S-MMT(P) and Bent(pH-s)), cytotoxicity level is reduced. On the other hand, nanoclays having large tactoids (S-MMT) and Bent) exhibit higher cytotoxicity due to more severe destruction of the cell membrane in the cell culture medium.

### 3.2. Mechanical Properties of the Neat PEVA, PEVA Nanocomposites and PEVA-DCN Nanocomposites Incorporating Physically Treated DCN (S-MMT(P)/Bent(pH-s))

Optimization of the mechanical properties of the PEVA-DCN nanocomposites can be gained when the best ratio of the S-MMT(P):Bent(pH-s) is used as a dual nanofiller. The tensile test is the most significant analysis for analyzing the mechanical performance of the PEVA-DCN nanocomposites with different S-MMT(P):Bent(pH-s) ratios. The best ratio of S-MMT(P):Bent(pH-s) can be determined by combining the mechanical test data with the supporting morphological data from SEM and TEM.

#### 3.2.1. Tensile Properties of Neat PEVA, PEVA Nanocomposites, and PEVA-DCN Nanocomposites

**Figure 4:**
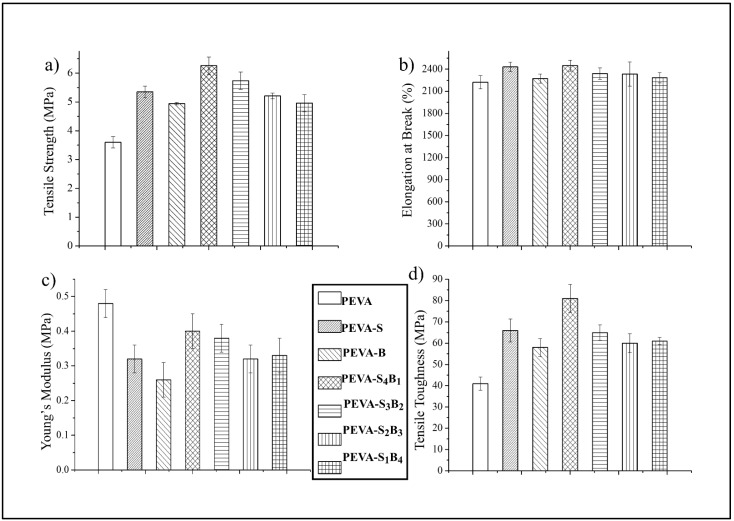
Tensile properties of neat PEVA, PEVA nanocomposites, and PEVA-DCN nanocomposites: (**a**) tensile strength (TS); (**b**) elongation at break (EB); (**c**) Young’s modulus (YM); (**d**) tensile toughness (TT).

The tensile properties of the neat PEVA, PEVA nanocomposites, and PEVA-DCN nanocomposite were analyzed. Figure 4a–d compare the tensile strength (TS), elongation at break (EB), Young’s modulus (YM), and tensile toughness (TT) of the materials, respectively. The mean values of all these data are tabulated in Table 2.

The tensile test data showed the enhancement in TS, EB, and TT of the PEVA copolymer when the S-MMT(P) and Bent(pH-s) were used as single filler and dual filler. This shows that both types of nanofiller are capable of improving the tensile properties of the copolymer. However, it is clearly seen that different ratios of S-MMT(P):Bent(pH-s) lead to different reinforcing capabilities. Therefore, the percentage of increment for TS, EB, and TT is seen to be changed by the ratio of both nanofillers in the PEVA copolymer structure.

Apparently, among all of the samples, PEVA-S_4_B_1_ exhibited the highest TS, EB, and TT values due to the inclusion of a well-dispersed DCN (S-MMT(P)/Bent(pH-s)) inside the PEVA copolymer matrix. The physical treatment weakened the van der Waals forces in the inter-galleries of both S-MMT and Bent, resulting in loosely packed silicate layers. Therefore, greater diffusion and intercalation of the polymeric chains in between the silicate layers occurred, increasing the contact surface area and interaction between the nanofiller and the matrix [24]. The well-bonded DCN/PEVA interface enabled the load transfer and energy dissipation in an area of high stress. As a result, significant improvement in the tensile strength and toughness of the PEVA matrix could be seen. TEM analysis reported in the next section proved that good dispersion and distribution of DCN could be obtained when the ratio of S-MMT(P):Bent(pH-s) was equal to 4:1 due to improved interactions between the nanofiller and the matrix.

In comparison with the neat PEVA, the TS, EB, and TT of the PEVA-S_4_B_1_ were enhanced by 74%, 10%, and 98%, respectively. The 40PEVA copolymer matrix consists of 60 wt% of the hydrophobic ethylene phase (non-polar) and 40 wt% of the hydrophilic vinyl acetate phase (polar). In this research study, S-MMT(P) and Bent(pH-s) were added as DCN for polarity matching with the PEVA copolymer matrix in order to encourage matrix–filler interactions in the resultant nanocomposite. S-MMT(P) nanofiller, which possesses hydrophobic characteristics, interacted with the ethylene phase while Bent(pH-s) (hydrophilic) approached the vinyl acetate phase. Hence, adding the most appropriate amount/ratio of polar/non-polar nanofiller into the PEVA may optimize the interaction forces and allow the greatest enhancement in the tensile properties of the copolymer.

Based on this result, it can be said that the PEVA-DCN nanocomposite sample with a high ratio of S-MMT(P) nanofiller produced a greater enhancement in tensile strength values compared to others. This is because the major phase of the PEVA copolymer is the hydrophobic polyethylene chains. Therefore, the high content of S-MMT(P) could encourage strong non-polar/non-polar interactions between the PEVA and the S-MMT(P) nanofiller. Furthermore, the S-MMT(P) nanofiller has greater interlayer spacing and less tactoid formation than the Bent(pH-s). As can be seen from the XRD graph pattern in Figure 2, the basal spacing (*d*_100_) of the S-MMT(P) (2.7 nm) was higher than Bent(pH-s) (1.6 nm) due to the presence of long alkyl chains of the organic surfactant that occupies the intergalleries of the nanoclay. This encourages the intercalation of S-MMT(P) nanoplatelets in between the polyethylene phase of the copolymer, including its crystalline region where a closed packed structure would inhibit the inclusion of larger platelets/tactoids of Bent(pH-s).

On the contrary, the incorporation of Bent(pH-s) as a single filler into the PEVA resulted in the smallest increment in the tensile strength of the copolymer. As seen in Table 2, the PEVA-B nanocomposite showed only 37% higher tensile strength than the neat PEVA. This was expected because the inclusion of ‘hydrophilic only’ nanofiller caused poor nanofiller–matrix interactions due to non-favorable interactions between the Bent(pH-s) and the hydrophobic polyethylene phase of the copolymer.

**Figure 5:**
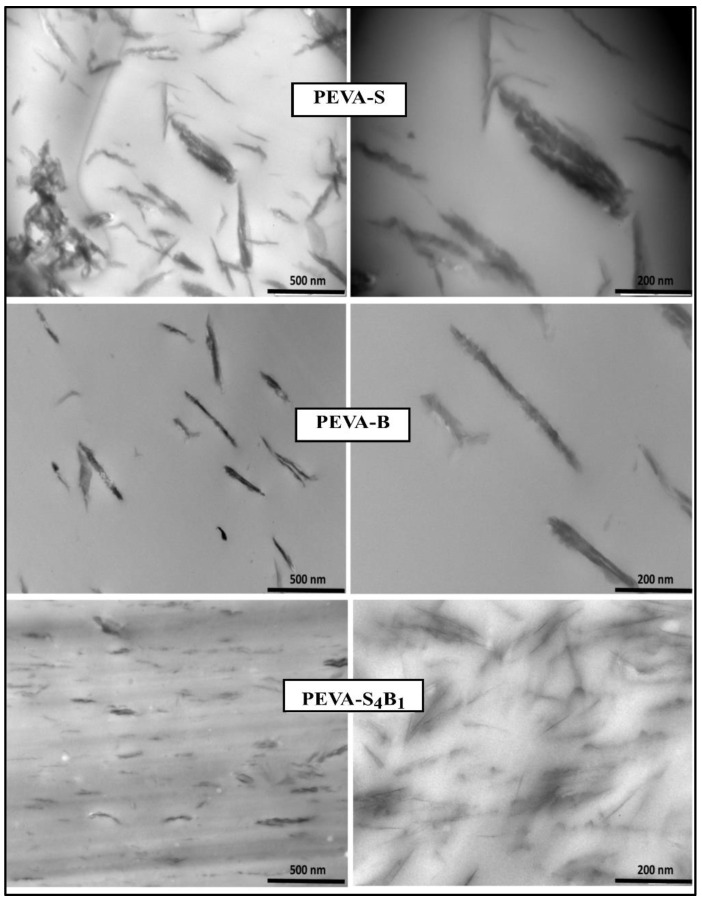
TEM micrograph of PEVA nanocomposites (PEVA-S and PEVA-B) and PEVA-DCN nanocomposites (PEVA-S_4_B_1_).

As seen in Figure 4c, a reduction in the Young’s modulus value of the copolymer occurred due to the inclusion of the single and dual nanofiller. All the nanocomposites (with single nanofiller and DCN) showed a lower Young’s modulus than the neat PEVA. Basically, there are two reasons why the modulus of host polymer can be decreased with the addition of nanofiller: The first reason is due to the poor matrix–nanofiller interactions that inhibit an efficient stress transfer mechanism from the matrix to the nanofiller. This scenario can be seen in the PEVA-B sample, where the nanocomposite showed much lower Young’s modulus than the neat PEVA. As seen in the TEM image (Figure 5) poor dispersion of the nanofiller in the PEVA matrix led to weak matrix–nanofiller interactions. The second reason is due to the plasticizing effect of the well-dispersed nanofiller to the matrix phase. Conformational freedom of the copolymer chains enhanced upon the intercalation of the clay nanoplatelets in between its molecular chains. This is because the nanofiller attachment on the PEVA copolymer matrix induces chain relaxation in the stress concentrated region, which allows a higher degree of copolymer chains conformation at the clay nanoclay–matrix interface. Thus, the elongation at break of the main polymer matrix can be enhanced because of this ‘plasticizing effect’, allowing a greater toughening mechanism of the matrix phase. This scenario can be seen, for example, in the PEVA-S_4_B_1_, where the Young’s modulus decrement was accompanied by the increment of the tensile toughness of the matrix.

Apparently, the results indicate that the PEVA-DCN nanocomposite that contained a high amount of S-MMT(P) possessed greater tensile properties compared to the others. The following TEM and SEM results support this finding.

#### 3.2.2. Dispersity Analysis of the PEVA Nanocomposites (PEVA-S and PEVA-B) and Optimum PEVA-DCN Nanocomposite (PEVA-S_4_B_1_)

TEM analysis was done on the PEVA nanocomposite and PEVA-DCN nanocomposite to verify the presence of intercalated and exfoliated structures of nanofillers (S-MMT (P) and Bent(pH-s)) in the copolymer matrix (PEVA). The TEM images are displayed in Figure 5. The PEVA-S_4_B_1_ sample was chosen since it possesses the best mechanical property data among other nanocomposites with dual clay nanofiller samples.

**Figure 6:**
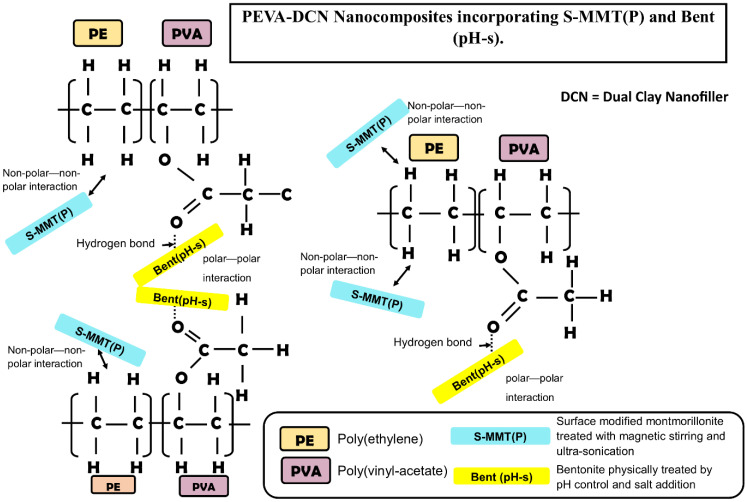
Interactions of dual nanofillers (S-MMT(P)): Bent(pH-s) with PE and PVA phase of PEVA copolymer chains.

The TEM image of the PEVA-DCN nanocomposite sample (PEVA-S_4_B_1_) signified the presence of well exfoliated and dispersed nanofillers in the PEVA copolymer matrix. A high amount of tiny and thin nano-size range particles were seen to be distributed evenly in the copolymer matrix. As proposed in Figure 6, hydrophobic S-MMT(P) nanofiller interacted well with the PE chains of the copolymer, while the hydrophilic Bent(pH-s) bonded well with the polyvinyl acetate (PVA) chains of the copolymer. These promoted good dispersion of the dual nanofiller in the PEVA copolymer matrix.

In contrast, the samples of PEVA-S and PEVA-B exhibited only small amounts of particles having large tactoids or lengthy aggregated platelets due to the low quality of nanofiller distribution and dispersion inside the matrix. In the case of the PEVA-S sample, it contained S-MMT nanofiller only, which had favorable interactions with the PE chains because it possessed the same hydrophobic characteristics; however, the nanofiller’s platelets were not easily intercalated by the hydrophilic PVA chains. In the case of PEVA-B sample, the Bent nanofiller had a greater affinity towards the PVA chains due to the similar hydrophilic characteristic. Hence, for both nanocomposite systems, insufficient interactions between the nanofiller and both PE and PVA phases of the copolymer matrix caused the nanofiller’s platelets to not be fully intercalated/dispersed well throughout the main matrix. As a consequence, poor dispersion and distribution of nanofillers in the PEVA copolymer matrix could be observed.

#### 3.2.3. Analyzing Tensile Fractured Surface by SEM

**Figure 7:**
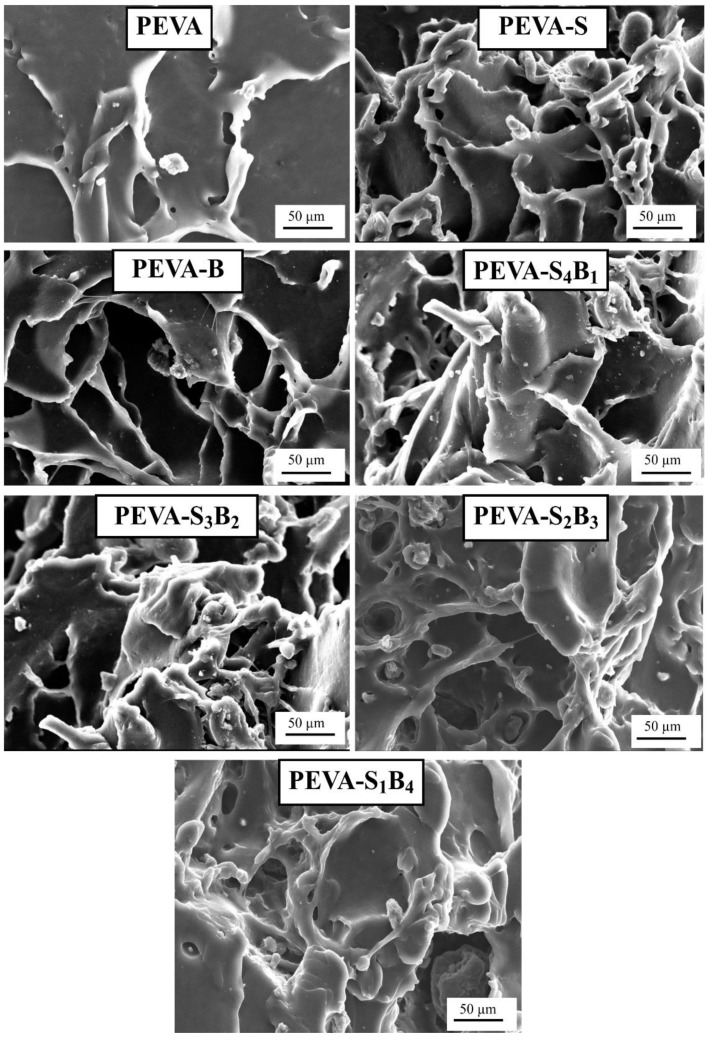
SEM micrograph of neat PEVA, PEVA nanocomposites (PEVA-S and PEVA-B), and PEVA-DCN nanocomposites (PEVA-S_4_B_1_, PEVA-S_3_B_2_, PEVA-S_2_B_3_, and PEVA-S_1_B_4_).

The tensile fracture surface was analyzed through SEM analysis, and the images are displayed in Figure 7. This analysis was performed to differentiate the fracturing behavior of the selected samples. SEM imaging was employed by many researchers to analyze fracture behavior after tensile tests [44,45,46]. Based on the appeared surface morphology, the PEVA nanocomposites (PEVA-S and PEVA-B) and PEVA-DCN nanocomposites (PEVA-S_4_B_1_, PEVA-S_3_B_2_, PEVA-S_2_B_3_, and PEVA-S_1_B_4_) samples exhibited more ductile fracture, showing longer fibrous surface morphology in comparison with the neat PEVA.

On the contrary, the neat PEVA showed a smaller amount of prominent fibrous surface, related to lower elongation at break, and high modulus of elasticity compared to PEVA nanocomposites and PEVA-DCN nanocomposite samples. The tensile fractured surface of the PEVA-S_4_B_1_ nanocomposite showed more significant matrix deformation upon the application of tensile load, suggesting that higher energy was absorbed through the PEVA molecular motions. Thus, upon the existence of the DCN, the enhancement of the values for tensile toughness and elongation at break was due to this matrix toughening mechanism.

### 3.3. Biostability Analysis

In this study, biostability analysis involved comparison of the morphology and mechanical properties of the neat PEVA, PEVA nanocomposites, and PEVA-DCN nanocomposites under ambient and in vitro conditions (in PBS solution at 37 °C for 3 months). Based on the above results, we can confirm that the S_4_:B_1_ comprises the best ratio of the dual nanofiller that can enhance the strength, flexibility, and toughness of the PEVA with 40 wt% of VA composition. Thus, the sample of PEVA-S_4_B_1_ was selected for the biostability analysis. The obtained data were compared with the control samples, which were neat PEVA, PEVA-S, and PEVA-B nanocomposites.

#### 3.3.1. Ambient and In Vitro Mechanical Properties by Tensile Test

**Figure 8:**
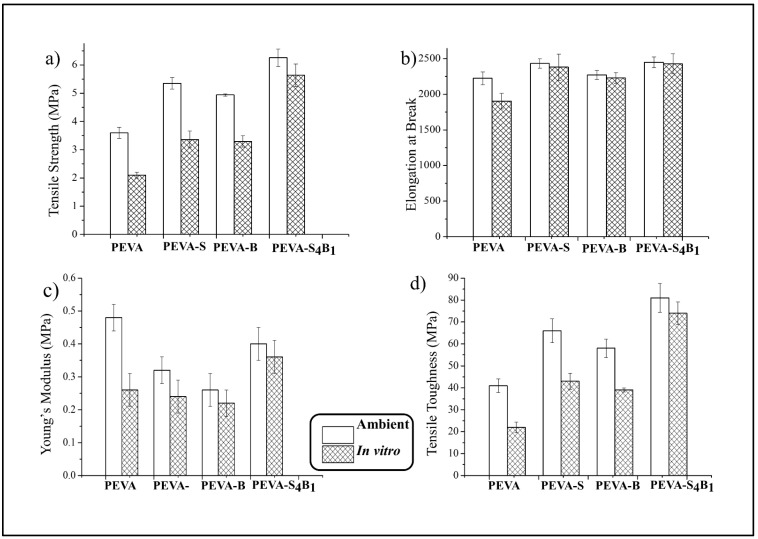
Comparison of the ambient and in vitro tensile properties of neat PEVA, PEVA nanocomposites, and PEVA-DCN nanocomposites: (**a**) tensile strength; (**b**) elongation at break; (**c**) Young’s modulus; (**d**) tensile toughness.

Tensile properties (TS, EB, YM, and TT) of the neat PEVA, PEVA nanocomposites, and PEVA-DCN nanocomposites (ambient and in vitro) are compared in Figure 8. Based on the results, it was noticeable that after exposure of the samples in the PBS solution for 3 months at 37 °C, the tensile properties of all the materials reduced. According to Lyu and Untereker [47], polymers used in biomedical devices are exposed to the human body temperature and physiological fluid, and thus their degradation may occur by hydrolysis, oxidation, and physical degradation processes. Permeability of the host copolymer to small quantities of the liquid/water vapor leads to a hydrolysis mechanism, causing degradation through the break-up of bonds and chains [2,48]. In fact, degradation kinetics can be enhanced when exposed to the human body temperature due to the acceleration of the hydrolysis process. In this study, all the samples were subjected to simulated body fluid (PBS, 37 °C) to mimic the human body environment; therefore, hydrolysis degradation was expected. Furthermore, the degradation process of the copolymer could also occur through the oxidation mechanism. The ions (H^+^ and OH^−^) from the PBS solution could react with oxygen molecules, producing more free radicals to accelerate the oxidation process. This is because the radicals can react with the copolymer molecular chains and can be transferred to other parts of the copolymer chains. Lastly, the biostability of the PEVA copolymer may also be reduced through the physical degradation process. In this case, the copolymer may undergo water-induced swelling, affecting its glass transition temperature (Tg), dimensional stability, and mechanical properties. Absorbed water can be said as a plasticizer to the polymer, reducing the Tg and rigidity of the polymer. It will also reduce the creep resistance of the copolymer. Consequently, the normal functions of the materials can be affected.

Among all the tested materials, the PEVA-S_4_B_1_ nanocomposite with DCN showed the best retention in TS, EB, YM, and TT (Figure 8) upon the 3 months of exposure in the in vitro conditions. The PEVA-S_4_B_1_ showed a reduction of about 10% (TS), 1% (EB), 10% (YM), and 9% (TT) only. In comparison, the neat PEVA possesses the decrement of TS, EB, YM, and TT of about 42%, 14%, 46%, and 46%, respectively. These reductions were much more significant compared to the PEVA-DCN nanocomposite sample. Therefore, it is clear that the addition of the dual nanofiller may reduce the degradation process through the above-mentioned mechanisms. This is because good interface bonding between the matrix and nanofiller may reduce the permeability of the water molecules and oxidative agents into the copolymer chains structure. Good dispersion and distribution of the dual nanofiller create a tortuous path for the entrance of these permeants and thus will resist the attack of both hydrolytic and oxidative agents on the copolymer chains. Furthermore, the more susceptible-to-degrade PVA phase of the copolymer that contains easy-to-hydrolyze non-carbon atoms can be protected by the Bent(pH-s) nanofiller through the developed polar–polar bonding between nanofiller and the PVA molecular chains. This is because the rigid structure of the Bent(pH-s) nanofiller can reduce the hydrolytic activity of the more vulnerable bonds of the PVA chains via steric hindrance. In addition, the platelets of Bent(pH-s) may also restrict the passage of fluid into the PVA chains.

Results also indicated that the PEVA nanocomposite with single filler (S-MMT(P) or Bent(pH-s)) showed much lower biostability than the optimum PEVA-DCN nanocomposite (PEVA-S_4_B_1_). The percentages of TS, EB, YM, and TT decrement were higher than those of the nanocomposite with a DCN. This is because weak matrix–nanofiller bonding and interactions induced the accumulation of water molecules at the interface of these two constituents, causing them to be separated further apart. This caused degradation by hydrolysis, oxidation, and physical processes to become faster.

#### 3.3.2. SEM Analysis (Surface Degradation after Exposure to the PBS Solution at 37 °C for 3 Months)

**Figure 9:**
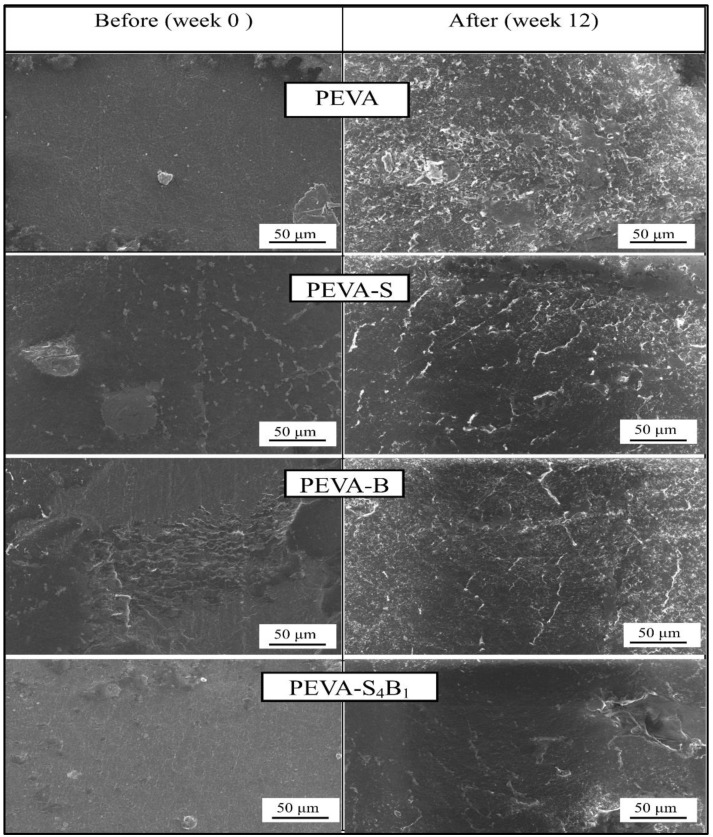
SEM images of neat PEVA, PEVA nanocomposites (PEVA-S and PEVA-B), and PEVA-DCN nanocomposites (PEVA-S_4_B_1_) before and after exposure to PBS fluids for 3 months at 37 °C.

Surface degradation of the neat PEVA, PEVA-B, PEVA-S, and PEVA-S_4_B_1_ was analyzed by comparing the surface morphology of the samples before and after 3 months of exposure in PBS solution at 37 °C. The SEM images captured for all samples are displayed in Figure 9.

Apparently, signs of degradation appeared in all samples upon 3 months of immersion in the PBS solution. Cracks and rough surfaces could be seen on the specimens of neat PEVA, PEVA-S, and PEVA-B. The neat PEVA sample before being exposed to the PBS solution had a smooth and homogeneous surface; however, it turned into a rough surface with cracks and voids after 3 months of exposure to the in vitro condition. As compared to the neat PEVA, the surface morphology of the PEVA-S and PEVA-B nanocomposites showed a lesser degree of surface degradation, smoother surface, and less cracks. As expected, the PEVA-S_4_B_1_ nanocomposite with a dual clay nanofiller exhibited the least degradation effect after being exposed to the in vitro condition for 3 months. These results were tallied with the biostability evaluation by the in vitro tensile test, where the most biostable system that could retain the tensile properties upon the in vitro treatment was found to be the PEVA-S_4_B_1_ nanocomposite. As mentioned earlier, the dual nanofiller helped to reduce the permeability of the host copolymer by creating a more tortuous path for the passage of the simulated body fluid. As a result, the degradation process through hydrolysis, oxidation, and water-induced swelling reduced significantly, producing more biostable copolymeric material.

## 4. Conclusions

Surface modified montmorillonite that underwent a physical treatment by magnetic stirring and an ultra-sonication process (S-MMT(P)) was combined with bentonite that underwent physical treatment by pH control and the salt addition process (Bent(pH-s)) to form a ‘dual clay nanofiller (DCN)’ for reinforcing and bio-stabilizing the PEVA copolymer for use in biomedical applications. FTIR results indicated that the chemistry aspect of both nanofillers was retained upon the physical treatment; however, the XRD data suggested that their basal spacings increased slightly due to the loosening of the original well-packed tactoids. A cytotoxicity assay suggested that the biocompatibility of both nanofillers was enhanced upon the physical treatment process. Tactoid size reduction and delamination reduced the cytotoxicity level of the nanoclay. Therefore, both nanofillers are safe to be used in low concentrations as reinforcing materials for the copolymer. The best ratio of S-MMT(P):Bent(pH-s) as a dual nanofiller was found to be 4:1. This determination was based on the tensile test data where the S_4_B_1_ dual nanofiller resulted in the highest achievement in tensile strength, elongation at break, and tensile toughness. TEM analysis proved that the PEVA containing S_4_B_1_ dual nanofiller (PEVA-S_4_B_1_) contained well dispersed and distributed particles throughout the matrix of the copolymer due to good interactions between the dual nanofiller and the PEVA matrix. Therefore, the biostability of the copolymer matrix was also enhanced significantly. The PEVA-S_4_B_1_ nanocomposite exhibited the best retention in tensile properties upon immersion of the sample in the simulated body fluid at 37 °C when benchmarked with the neat PEVA and other nanocomposite samples. Furthermore, surface degradation also appeared to be much less. These findings demonstrate the potential of PEVA-DCN nanocomposites for biomedical applications.

## Figures and Tables

**Table 1:** The formulation of PEVA nanocomposites and PEVA-DCN nanocomposites containing S-MMT(P)/Bent(pH-s) dual nanofiller.

Type of Samples	Acronym	Weight (%)		
		PEVA	S-MMT (P)	Bent (pH-s)
Neat PEVA	PEVA	100	-	-
PEVA + S-MMT(P)	PEVA-S	96	4	-
PEVA + Bent(pH-s)	PEVA-B	96	-	4
PEVA + 3.2 wt% S-MMT(P) + 0.8 wt% Bent(pH-s)	PEVA-S_4_B_1_	96	3.2	0.8
PEVA + 2.4 wt% S-MMT(P) + 1.6 wt% Bent(pH-s)	PEVA-S_3_B_2_	96	2.4	1.6
PEVA + 1.6 wt% S-MMT(P) + 2.4 wt% Bent(pH-s)	PEVA-S_2_B_3_	96	1.6	2.4
PEVA + 0.8 wt% S-MMT(P) + 3.2 wt% Bent(pH-s)	PEVA-S_1_B_4_	96	0.8	3.2

**Table 2:** Tensile strength, elongation at break, modulus of elasticity, and tensile toughness of neat PEVA, PEVA nanocomposites, and PEVA-DCN nanocomposites.

Type of Sample	Tensile Strength (TS) (MPa)	Elongation at Break (EB) (%)	Young’s Modulus (YM) (MPa)	Tensile Toughness (TS) (MPa)
PEVA	3.60 ± 0.2	2224 ± 89	0.48 ± 0.04	41 ± 3.1
PEVA-S	5.35 ± 0.2	2431 ± 64	0.32 ± 0.04	66 ± 5.4
PEVA-B	4.94 ± 0.04	2272 ± 63	0.26 ± 0.05	58 ± 4.2
PEVA-S_4_B_1_	6.26 ± 0.3	2448 ± 75	0.40 ± 0.05	81 ± 6.6
PEVA-S_3_B_2_	5.74 ± 0.3	2341 ± 76	0.38 ± 0.04	65 ± 3.6
PEVA-S_2_B_3_	5.21 ± 0.1	2335 ± 164	0.32 ± 0.04	60 ± 4.5
PEVA-S_1_B_4_	4.96 ± 0.3	2284 ± 71	0.33 ± 0.05	61 ± 1.7

## Data Availability

The data presented in this study are available on request from the corresponding author.
